# An Explanation of the Real Process of the "Spontaneous Evolution of the Fœtus," with Some Remarks, &c. &c.

**Published:** 1819-07-01

**Authors:** 


***An Explanation of the real Process of the "Spontaneous Evolution of the Fœtus," with some Remarks, &c. &c.*** By JOHN DOUGLAS, M. D. Licentiate of the College of Physicians, Ireland. Octavo. pp. 45, 1819.

Dr. DOUGLAS has witnessed seven cases of the spontaneous evolution of the fœtus, and his view of the *modus operandi* of nature, in such instances, is far more intelligible to us than Dr. Denman's theory, which every one is acquainted with. All practitioners acknowledge that shortly previous to the evolution, the shoulder of the child is forced very low in the pelvis, and the thorax occupies so much of its cavity as to preclude the practicability of passing the hand into the uterus. The parts abovementioned, instead of *receding* into the uterus, are, at each successive pain, forced still lower, until the ribs of that side corresponding with the protruded arm, press on the perinæum, and cause it to assume the form of the forehead in a natural labour. At this period the arm and shoulder can be perceived externally. By further uterine contractions the ribs are forced more forward, appearing at the os externum. The entire of the fœtus now resembles the larger segment of a circle; the head rests on the pubis internally; the clavicle presses against the pubis externally; with the acromion stretching towards the *mons veneris:* the breech is either in the hollow of the sacrum or at the brim of the pelvis, ready to descend into it. By a few further uterine efforts, the remainder of the trunk, with the lower extremities, is expelled.

In respect to the arm originally protruded, Dr. D. affirms that not one line of it or any other part once descended, ever is withdrawn again into the uterus. The arm and shoulder merely go more forward on the symphysis pubis externally. Such is precisely the process minutely observed in seven different cases. In no instance did the process require more than six hours. The infants were, of course, still born, and must be so. The followiug are the circumstances which would induce our author to prognosticate a spontaneous evolution, and a safe result. "If the arm of the fœtus should be almost entirely protruded, with the shoulder pressing on the perinæum—if a considerable portion of its thorax be in the hollow of the sacrum, with the axilla low in the pelvis—if, with this disposition, the *uterine* efforts be still powerful, and if the thorax be forced sensibly lower during the presence of each successive pain, the evolution may be expected with great confidence." P. 43.

There are many valuable hints in this little pamphlet, which w recommend to the attention of our obstetric brethren.

